# Common Dysregulation of Innate Immunity Pathways in Human Primary Astrocytes Infected With Chikungunya, Mayaro, Oropouche, and Zika Viruses

**DOI:** 10.3389/fcimb.2021.641261

**Published:** 2021-03-15

**Authors:** Victor Emmanuel Viana Geddes, Otávio José Bernardes Brustolini, Liliane Tavares de Faria Cavalcante, Filipe Romero Rebello Moreira, Fernando Luz de Castro, Ana Paula de Campos Guimarães, Alexandra Lehmkuhl Gerber, Camila Menezes Figueiredo, Luan Pereira Diniz, Eurico de Arruda Neto, Amilcar Tanuri, Renan Pedra Souza, Iranaia Assunção-Miranda, Soniza Vieira Alves-Leon, Luciana Ferreira Romão, Jorge Paes Barreto Marcondes de Souza, Ana Tereza Ribeiro de Vasconcelos, Renato Santana de Aguiar

**Affiliations:** ^1^ Laboratório de Virologia Molecular, Departamento de Genética, Instituto de Biologia, Universidade Federal do Rio de Janeiro, Rio de Janeiro, Brazil; ^2^ Laboratório de Biologia Integrativa, Departamento de Genética Ecologia e Evolução, Instituto de Ciências Biológicas, Universidade Federal de Minas Gerais, Belo Horizonte, Brazil; ^3^ Laboratório de Bioinformática, Laboratório Nacional de Computação Científica, Ministério de Ciência Tecnologia e Comunicações, Petrópolis, Brazil; ^4^ Instituto de Microbiologia Paulo de Goes, Universidade Federal do Rio de Janeiro, Rio de Janeiro, Brazil; ^5^ Instituto de Ciências Biomédicas, Universidade Federal do Rio de Janeiro, Rio de Janeiro, Brazil; ^6^ Departamento de Biologia Celular e Molecular, Faculdade de Medicina de Ribeirão Preto, Universidade de São Paulo, Ribeirão Preto, Brazil; ^7^ Hospital Universitário Clementino Fraga Filho, Universidade Federal do Rio de Janeiro, Rio de Janeiro, Brazil

**Keywords:** Chikungunya, Mayaro, Oropouche, Zika, innate immunity, astrocytes

## Abstract

Arboviruses pose a major threat throughout the world and represent a great burden in tropical countries of South America. Although generally associated with moderate febrile illness, in more severe cases they can lead to neurological outcomes, such as encephalitis, Guillain-Barré syndrome, and Congenital Syndromes. In this context astrocytes play a central role in production of inflammatory cytokines, regulation of extracellular matrix, and control of glutamate driven neurotoxicity in the central nervous system. Here, we presented a comprehensive genome-wide transcriptome analysis of human primary astrocytes infected with Chikungunya, Mayaro, Oropouche, or Zika viruses. Analyses of differentially expressed genes (DEGs), pathway enrichment, and interactomes have shown that Alphaviruses up-regulated genes related to elastic fiber formation and N-glycosylation of glycoproteins, with down-regulation of cell cycle and DNA stability and chromosome maintenance genes. In contrast, Oropouche virus up-regulated cell cycle and DNA maintenance and condensation pathways while down-regulated extracellular matrix, collagen metabolism, glutamate and ion transporters pathways. Zika virus infection only up-regulated eukaryotic translation machinery while down-regulated interferon pathways. Reactome and integration analysis revealed a common signature in down-regulation of innate immune response, antiviral response, and inflammatory cytokines associated to interferon pathway for all arboviruses tested. Validation of interferon stimulated genes by reverse transcriptase quantitative polymerase chain reaction (RT-qPCR) corroborated our transcriptome findings. Altogether, our results showed a co-evolution in the mechanisms involved in the escape of arboviruses to antiviral immune response mediated by the interferon (IFN) pathway.

## Introduction

Arthropod-borne viruses (*i.e*. arboviruses) comprehend a large group of viruses belonging to different families that are transmitted mainly by the bite of infected mosquitoes or ticks during the blood meal. As many of them are the etiological agents of relevant human pathologies, they pose a special threat in vulnerable countries around the world. Tropical countries of South America, such as Brazil, are specially in risk for arboviruses outbreaks due to the suitable warm weather, abundance of insect vectors, deforestation, high population concentration near forest areas, social-economical disparities, and overburden of the public healthcare system (Peña-García et al., 2017; Figueiredo, 2019; Medeiros and Vasconcelos, 2019). Many endemic outbreaks along the history, as well the recent introduction of arboviruses in Brazil, have risen the alarm of scientific and medical communities concerning the risk of new outbreaks, co-circulation of those viruses, and increasing of more severe cases (Metsky et al., 2017; Zanotto and Leite, 2018; Lorenz et al., 2019; Gutierrez et al., 2020). Viruses members of *Alphavirus*, *Orthobunyavirus*, and *Flavivirus* genus (*Togaviridae*, *Peribunyaviridae*, and *Flaviviridae* viral families, respectively) present a higher incidence in Brazil and has been associated to a plethora of clinically relevant outcomes, such as mild to debilitating fever, hemorrhagic fever, arthritis, microcephaly, and neurological disorders (Figueiredo, 2015; Santiago et al., 2015; Burt et al., 2017; Azeredo et al., 2018; Vernal et al., 2019; Brito Ferreira et al., 2020; Diagne et al., 2020; Gutierrez et al., 2020).

Alphaviruses as Chikungunya Virus (CHIKV) and Mayaro Virus (MAYV) circulate in Brazil, with many cases reported from Northern to Southeastern regions of the country (Carvalho et al., 2019; Lorenz et al., 2019; Naveca et al., 2019; Vasconcellos et al., 2019). They are enveloped viruses with single positive RNA (+) strand genome, flanked by 5’ and 3’ UTRs (untranslated regions). The genome is ~11.5 kb long and composed by two ORFs (open reading frame): the first one encodes the non-structural proteins nsP1-4 that plays a role in viral replication and host factors interaction and modulation, followed by a second ORF that encodes the structural proteins C (capsid), E3, E2, E1 (envelope glycoproteins) (Acosta-Ampudia et al., 2018; Frolov and Frolova, 2019; Levi and Vignuzzi, 2019). CHIKV are mainly transmitted by mosquito vectors from genera *Aedes* and MAYV is more restricted to sylvatic species of *Sabethes* and *Haemagogus*, reflecting on the epidemic pattern of CHIKV and in local MAYV circulation. Those alphaviruses cause a febrile illness, with common symptoms such as nausea, headache, rash, myalgia, and polyarthralgia. The arthralgia can persist for months after the acute infection, characterizing the main chronic incapacitating symptom of those infections. However, both viruses have been associated with more severe neurological manifestations, such as meningo-encephalitis, Guillain-Barré syndrome, acute disseminated encephalomyelitis (ADEM), and severe myelitis, with increasing reports of neuro-CHIKV in endemic areas such as Brazil (Bandeira et al., 2016; Balavoine et al., 2017; Khatri et al., 2018; Maria et al., 2018; Simon et al., 2018; Hameed and Khan, 2019; Silva et al., 2020).

Oropouche Virus (OROV) is an *Orthobunyavirus* responsible for many outbreaks, in Brazil and other South America countries (Elliott, 2014; De Regge, 2017). OROV genome is composed of three single strand negative RNAs (−) flanked by 5’ and 3’ UTRs: namely the S (small) RNA, a 961 nt long RNA that encodes the nucleocapsid protein N and the non-structural protein NSs; a 4.5 kb long M (medium) RNA that encodes the viral envelope glycoproteins Gn and Gc, and the non-structural protein NSm; and a 6.9 kb long L (large) RNA that encodes the viral RNA-dependent RNA polymerase (RdRp) (Travassos da Rosa et al., 2017; Sakkas et al., 2018). The midge *Culicoides paraensis* has been considered the main urban vector of OROV, although it was already isolated from other mosquitoes’ vectors, including species from the genera *Aedes* and *Culex*. OROV is the causative agent of Oropouche Fever, a mild febrile illness that shares common symptoms with other arboviral fevers as moderate to high fever, headache, nausea and diarrhea, vomiting, dizziness, and myalgia. Photophobia is also a particular common symptom and, in some cases, OROV has been associated with neurological meningo-encephalitis outcomes (Bastos et al., 2012; Santos et al., 2014; Vernal et al., 2019).

Zika Virus (ZIKV) has raised global attention in the past few years as a potential threat to humans, mainly to pregnant women, due to the infection could promote severe neurological disorders and congenital fetal malformations in Brazil (Duffy et al., 2009). Those clinical outcomes were first described in the last outbreaks, in French Polynesia (2013) and with the arrival and spread of ZIKV throughout the Americas (2014) (Cao-Lormeau et al., 2016; Schuler-Faccini et al., 2016). ZIKV is a flavivirus with a genome composed of a single positive strand RNA of ~11 kb, flanked by 5’ and 3’ UTR regions coding a precursor polyprotein that is processed by viral and cellular protease to generate three structural proteins C (capsid), prM, and E (envelope) and seven non-structural proteins NS1, NS2A, NS2B, NS3, NS4A, NS4B, and NS5. The epidemics in South America between 2014 and 2016 brought light to the severe neurological manifestations of ZIKV infection, named Congenital Zika Syndrome (CZS), that affects the nervous system and multiple organs of the developing fetus of infected pregnant women. The CZS comprehend a myriad of clinical neurological manifestations including microcephaly, hydrocephaly and lissencephaly as well as ventricular calcifications and arthrogryposis (congenital joints contracture) (Liang et al., 2019). In adults, ZIKV infection can also be associated with encephalitis, ADEM, and Guillain-Barré Syndrome (Bautista, 2019).

Despite all the description of neurological associated manifestations of arboviruses, the neurotropism of viruses such as MAYV and OROV is still elusive. CHIKV and ZIKV can infect central nervous system (CNS) cells such as: neurons, astrocytes, and microglia (Klein et al., 2019). Astrocytes play a pivotal role in the CNS and are highly associated with encephalitis caused by those viruses (Soung and Klein, 2018). Astrocytes are responsible for the maintenance of the Blood-Brain Barrier (BBB) interacting with the brain microvascular endothelial cells (BMECs) (Cardoso et al., 2010), secretion of proinflammatory cytokines (Voet et al., 2019), recruitment of T cells upon brain injury or infection (Zhang et al., 2008), regulation of synapses, and regulation of glutamate uptake (Olsen et al., 2015). Viral infections can induce apoptosis in neuronal cells and dysregulate astrocyte functions increasing the BBB permeability and neuroinvasion by up-regulating metalloproteinases expression (Wang et al., 2008; Ju et al., 2009; Yang et al., 2012), promoting injury derived from long-term astrocytic inflammatory response (Garber et al., 2018), and neurotoxicity by accumulation of extracellular glutamate (Cisneros and Ghorpade, 2012), which all together contributes to the encephalitis process.

Here, we present a comparative transcriptome study of human primary astrocytes cells infected with CHIKV, MAYV, OROV, or ZIKV describing the specific genes and pathways modulated by each virus and the integration of common pathways by system biology strategy. Human primary astrocytes were highly permissive to all viruses with consequent down-modulation of IFN and interferon-stimulated genes (ISGs) pathways as a viral escape strategy to the innate antiviral immune response.

## Materials and Methods

### Primary Human Astrocyte Isolation

Adult primary human astrocytes were isolated from surgically resected anterior temporal lobe tissue, from patients selected for surgical treatment of temporal lobe epilepsy associated with hippocampal sclerosis. The selected patients were evaluated by video-electroencephalography monitoring with a 132-channel Nihon-Kohden^®^ apparatus, and the ictal onset zone was concordant with neuroimaging and semiology data. The pathological tissue targeted in surgery for these cases is the gliotic hippocampus, and the anterior temporal lobe resection is used merely as a surgical pathway to the diseased area. All patients gave written consent to the study, and the procedures were in agreement with the Brazilian Ministry of Health Ethics Committee (Certification of Presentation for Ethical Approval, *CAAE*, submission number 69409617.9.0000.5258, decision number 2.792.114). As previously described (Sebollela et al., 2012), only healthy cortical tissue was used to produce astrocyte cultures. Experimental protocols were performed as described previously (Diniz et al., 2012; Dezonne et al., 2017). Briefly, tissues were washed in Dulbecco’s Modified Eagle’s Mediu (DMEM), mechanically dissociated, chopped into small (<2 mm3) pieces with a sterile scalpel, and incubated in 10 ml of 0.25% trypsin solution at 37°C for 10 min. After centrifugation for 10 min, the cell pellet was resuspended in DMEM/F-12 growth medium supplemented with 10% Fetal Calf Serum (FCS) and plated onto tissue culture plates in a humidified 5% CO_2_, 95% air atmosphere at 37°C for 2 h in order to achieve adherence of microglial cells. The non-adherent astrocytes were transferred to other culture plates, previously coated with poly-L-lysine. Adherent astrocytes were allowed to grow by replacing the medium once a week. New passages of cells were generated by harvesting confluent astrocyte cultures using trypsin-EDTA (Ethylenediamine tetraacetic acid) solution (0.25% trypsin with EDTA; *Thermo Fisher Scientific*). Human astrocytes from up to the seventh passage and from two different donors were used in the study.

### Cell Lines, Viruses, and Infections

Vero cells (*ATCC*, *CCL-81*) were maintained in DMEM (*Gibco*) and primary astrocytes were maintained in DMEM/F12 (*Gibco*), both supplemented with 10% v/v Fetal Bovine Serum (FBS) (*Gibco*) and 1% v/v of penicillin-streptomycin (10.000 U/ml-10.000 µg/ml) (*Gibco*). DMEM/F12 was also supplemented with 1% v/v of glutamine (200 mM, *Gibco*), 0.6% g/v of glucose (*Sigma-Aldrich*), and 0.13% g/v of sodium bicarbonate (*Sigma-Aldrich*). All cells were incubated at 37°C and 5% CO_2_. CHIKV isolate BHI3745/H804709 (GenBank accession #KP164570.1), MAYV strain 4675 (*ATCC 66*, GenBank accession #MK070492.1), OROV strain BeAn19991 (GenBank accession #KP052852.1, #KP052851.1, #KP052850.1), and ZIKV strain PE/243 (GenBank accession #KX197192.1) were used to perform infection. Viral stocks used here are expressed as Plaque Forming Units/ml (CHIKV, 1.6 × 10^8^ PFU/ml; MAYV, 7 × 10^7^ PFU/ml; OROV, 2 × 10^6^ PFU/ml; and ZIKV, 2.6 × 10^6^ PFU/ml) and were propagated by serial passages in Vero cells by routine methods using DMEM. Astrocyte infections were performed at multiplicity of infection (MOI) 1 (2 × 10^5^ cells/well) for all four viruses in 12-well plate during 1 h at 37°C and 5% CO_2_ in medium without FBS, under biosafety level 3 conditions at a Biosafety Level-3 laboratory at Federal University of Rio de Janeiro. For each infection, uninfected cells from the same donor were used in a pairwise experiment. Virus titration was performed by plaque assay in Vero cells plated at 3 × 10^5^ cells/well in 12-well plates 1 day prior to infection. After 1 h incubation with the virus, cells medium was replenished by DMEM supplemented with 1% v/v FBS, 1% v/v antibiotics, and 1% v/v carboxymethyl cellulose (CMC) (*Sigma-Aldrich*), and incubated at 37°C and 5% CO_2_ during 2 days for CHKV and MAYV, and 5 days for OROV and ZIKV. Cells were fixed with 4% formaldehyde for 20 min at room temperature, washed in Phosphate Buffered Saline (PBS) (*Gibco*), and stained with 20% v/v ethanol-violet crystal solution for 15 min for visual counting. Astrocytes viability assay upon virus infection was evaluated by CellTiter-Blue (*Promega*) according to manufacturer’s instructions. The fluorescence was measured at SpectraMax Paradigm Multi-Mode Detection Platform (*Molecular Devices*).

### Flow Cytometry, Immunofluorescence, and Antibodies

Virus infectivity was evaluated using specific antibodies against viral proteins as follows. Briefly, astrocytes (2 × 10^5^ cells/sample) were fixed with 4% paraformaldehyde for 20 min and permeabilized in 1% v/v Triton X-100 PBS solution. Blocking was performed in 5% v/v Donkey Serum (*Sigma-Aldrich)* PBS solution for 1h at 37°C. Primary antibodies for each virus were diluted as follows: for CHIKV was used 1:50 of mouse monoclonal J31F (*Thermo Fisher Scientific*), for MAYV was used 1:300 of mouse monoclonal anti-alphavirus E1 antibody (MAB8754, *Sigma-Aldrich*), for OROV was used 1:300 of mouse polyclonal anti-OROV antibody, and for ZIKV was used 1:300 of mouse monoclonal anti-NS1 antibody (Abcam, ab218546), all diluted in blocking solution at 37°C for 30 min. Cells were then washed thrice in PBS and incubated with 2 µg/ml *Donkey anti-mouse AlexaFluor 488* secondary antibody (*Thermo Fisher Scientific*) at 37°C for 30 min. After incubation with the secondary antibody, cells were washed and resuspended in PBS. Flow cytometry was performed using Accuri C6 cytometer (*BD Biosciences*). At least 10,000 gated events were counted per experimental replica at Fluorescein isothiocyanate (FITC) channel. Astrocytes specific markers were confirmed by immunofluorescence. For that, cells were fixed and permeabilized as described above, followed by blocking in PBS 3% Bovine Serum Albumin (BSA) (*Sigma-Aldrich*) and 5% Normal Goat Serum (NGS) (*Thermo Fisher Scientific*) for 1 h and followed by overnight incubation at 4°C with the primary antibodies. Primary antibodies were diluted in block solution as follows: 1:500 of rabbit anti-GFAP (Glial Fibrillary Acidic Protein) (*Dako, Glostrup, DK*), 1:200 of mouse anti-GS (Glutamine Synthetase) (*Merck-Milipore*), 1:200 of rabbit anti-GLAST(Glutamate Aspartate Transporter 1) (*Abcam*), and 1:200 of rabbit anti-GLT1 (Glutamate Transporter 1) (*Abcam*). Cells were washed with PBS and incubated for 2 h with 1:400 dilution of goat anti-rabbit or anti-mouse IgG *AlexaFluor 488*, or 1:1,000 dilution of goat anti-rabbit IgG *AlexaFluor 546* secondary antibodies (*Thermo Fisher Scientific*). Nuclei was stained with 4’,6-Diamidino-2-Phenylindole, Dihydrochloride (DAPI) (*Sigma-Aldrich*). Cells were mounted with mounting medium (DakoCytomation) and imaged under a confocal microscope (Leica TCS SPE).

### RNA Isolation, Quality Assessment, and RNA-Seq

Astrocytes were plated at 2 × 10^5^ cells/well and infected at MOI 1 with either CHIKV, MAYV, OROV, or ZIKV. Eighteen h post-infection (hpi) for CHIKV, MAYV, and OROV or 48 hpi for ZIKV, cells were washed with PBS, trypsinized, centrifuged, and the pellet were used for RNA extraction. Six replicates of non-infected astrocytes and three replicates of infected astrocytes were used for each virus. Total cellular RNA was isolated using *RNeasy Mini kit* (*Qiagen*) according to manufacturer’s instructions. RNA quantification was performed using *Qubit RNA HS Assay kit* and *Qubit 3 Fluorometer* (*Thermo Fisher Scientific*). Genomic DNA contamination was avoided by DNase treatment (TURBO DNA-free Kit, *Thermo Fisher Scientific*) before RNA-Seq and RT-qPCR validation. Only samples with *RNA integrity number* (RIN) ≥ 9 were used, as verified by *RNA 6000 Pico Kit* and *2100 Bioanalyzer* (*Agilent Technologies*). Ribossomal RNA was depleted using *Ribo-Zero Gold* (*Illumina*) and 200 ng of total RNA for each sample were used for library preparation with TruSeq Stranded Total RNA Library Prep (*Illumina*) according to manufacturer’s instructions. RNA-seq were performed in 2 × 9 samples using *NextSeq 500/550 High Output v2 Kit (150 cycles)* (*Illumina*) in a NextSeq 550 platform (*Illumina*).

### RNA-Seq Statistical Analysis

The RNA sequence files (fastq) were applied to the software BBDuk2 (https://jgi.doe.gov/data-and-tools/bbtools/bb-tools-user-guide/bbduk-guide) to trim low quality-related sequences (bellow Q30) and remove Illumina adapter-contaminant tags. The FASTQC (https://www.bioinformatics.babraham.ac.uk/projects/fastqc) reports the overall quality of the sequencing. The aligner HiSat2 (Kim et al., 2015) mapped the filtered reads to the human genome assembly GRCh38.p12 presented at the Ensembl database (https://www.ensembl.org/Homo_sapiens). The R/Bioconductor package Rsubread using the function of feature counts (Liao et al., 2019) performed the counting table of the mapped reads for the following statistical analysis. The R/Bioconductor package DESeq2 (Love et al., 2014) conducts the differential gene expression (DGE) test. It is also applied to the R/Bioconductor package apeglm (Zhu et al., 2019) to shrink log-fold change. The criteria of the adjusted p-value (corrected by false discovery rate—FDR) below 0.05 and log2 fold change (FC) above the absolute value of 1.0 was followed to classify genes as differentially expressed. The GOstats (Falcon and Gentleman, 2007) performed the Gene Ontology (GO) enrichment analysis. The package Pathview (Luo and Brouwer, 2013) visualizes the KEGG pathways and shows the associated genes. The ReactomePA package (Yu and He, 2016) leads the Reactome database analysis applying enrichment and allowing to create graphics. All the results and additional information about the RNAseq analysis were stored in the relational database PostgreSQL 12. The data can be easily accessed at the site: http://biotools.labinfo.lncc.br/astrocitovirus_data. The dataset used in our study is publicly available in SRA-NCBI (www.ncbi.nlm.nih.gov/sra), SRA accession PRJNA662366.

### RT-qPCR Validation

Cells were seeded (2 × 10^5^ cells/sample) and infected at MOI 1 as described above. The RNA was extracted and treated as described above at the indicated times points post infection and cDNA was produced using High Capacity cDNA Reverse Transcription Kit (*Thermo Fisher Scientific*) and 500 ng of RNA. Quantitative PCR was performed in three biological replicates per condition using 20 ng of RNA input and *SYBR Green PCR Master Mix* (*Applied Biosystems*) on 7500 Fast Real-Time PCR System (*Thermo Fisher Scientific*) according to manufacturer’s instructions. Hypoxanthine Phosphoribosyltransferase 1 (*HPRT1*) gene was used as normalization and pre-designed *PrimeTime* primers (*Integrated DNA Technologies*) were used for selected target genes according to manufacturer’s instruction (for primers sequences see **Table S1**). Normalized gene expression was compared between experimental and mock non-infected conditions using Mann-Whitney test. Fold change and p-value for each comparison are presented using volcano plots.

## Results

### Human Primary Astrocytes Are Permissive to Different Arboviruses Infection

**Figure 1:**
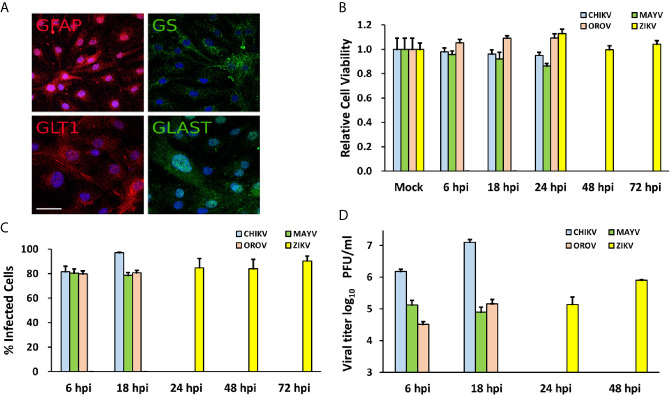
Human primary astrocytes are permissive to CHIKV, MAYV, OROV, and ZIKV infection. Human primary astrocytes were isolated from brain surgery and infected with CHIKV, MAYV, OROV, or ZIKV at MOI 1 in different time points. **(A)** Immunofluorescence microscopy showing astrocytes specific cell markers: GFAP, Glial Fibrillary Acidic Protein, and GLT1, Glutamate Transporter 1 (both in red); GS, Glutamine Synthetase, and GLAST, Glutamate Aspartate Transporter 1 (both in green). Nuclei are stained with DAPI (cyan). Scale bar = 50 µm. **(B)** Cell viability post infection was measured at indicated time points. All infected cells were normalized by mock (uninfected) cells. **(C)** Virus infectivity was measured by flow cytometry using primary antibodies for virus proteins: CHIKV (J31F, anti-E1 antibody); MAYV (anti-alphavirus E1 antibody); OROV (anti-OROV serum), and ZIKV (anti-NS1 antibody) and plotted as percentage of infected cells in gated population. 10.000 events were recorded. **(D)** Viral production measured by plaque assay from the supernatants of infected cells. Viral titers were plotted as log_10_ Plaque Forming Units/ml. All the experiments were performed on three replicates from two independent experiments presented here as media and standard deviation (SD).

In order to investigate the neuropathogenesis of arboviruses, we infected human cortical primary astrocytes (hAST) with CHIKV, MAYV, OROV, or ZIKV due to those cells multiple roles in maintaining the homeostasis of CNS and respond to infections. We confirmed the expression of classical astrocytes markers such as: glial protein (GFAP), glutamine synthetase (GS), and glutamate transporters (GLT1 and GLAST); which corroborates the same previously demonstrated astrocytic phenotype derived from induced pluripotent stem cells (iPSC) (**Figure 1A**) (Sebollela et al., 2012; Diniz et al., 2014; Garcia et al., 2014; Dezonne et al., 2017; Eugenio et al., 2018; da Silva et al., 2019). No cytopathic effects were observed in response to CHIKV, MAYV, and OROV until 48 h post infection (hpi) and until 72 h post ZIKV infection (**Figure 1B**). We also assured that at least 80% of cells were infected in that time span at MOI 1, as demonstrated by flow cytometry using antibodies against each virus protein (**Figure 1C**). Flaviviruses as ZIKV presents a slow replication rate compared to alphaviruses such as CHIKV and MAYV, taking more time to reach the same levels of replication. Even in the earliest time point (i.e., 6 hpi for CHIKV, MAYV, and OROV, and 24 hpi for ZIKV) 80% of cells were infected with all viruses tested, reinforcing that hAST is highly susceptible to different arboviruses infections, including MAYV and OROV, which have never been described before. To assure that infections were productive in astrocytes cells, we quantified the viral titer derived from the supernatant of infected cells. All arboviruses were able to produce infectious particles as evaluated by plaque assay (**Figure 1D**). Alphaviruses infection were more productive in those cells, followed by Orthobunyavirus and Flavivirus, respectively. CHIKV produced the highest titer: 1.52 × 10^6^ PFU/ml as soon as 6 hpi, peaking 1.24 × 10^7^ PFU/ml at 18 hpi; followed by MAYV and OROV, respectively. Again, ZIKV took additional 24 h to reach the same levels of virus production compared to the other viruses, confirming its lower replication rate in hAST compared with CHIKV, MAYV, and OROV (**Figure 1D**). With exception of ZIKV (Hamel et al., 2017; Kozak et al., 2017; Meertens et al., 2017; Chen et al., 2018; Stefanik et al., 2018; Sher et al., 2019), this is the first study to demonstrate the capacity of CHIKV, MAYV, and OROV to infect hAST, corroborating their potential as agents of neuro-associated outcomes.

### Alphaviruses (CHIKV and MAYV) Replicate Better in Human Astrocytes Cells and Modulate a Higher Number of Cellular Genes

**Figure 2:**
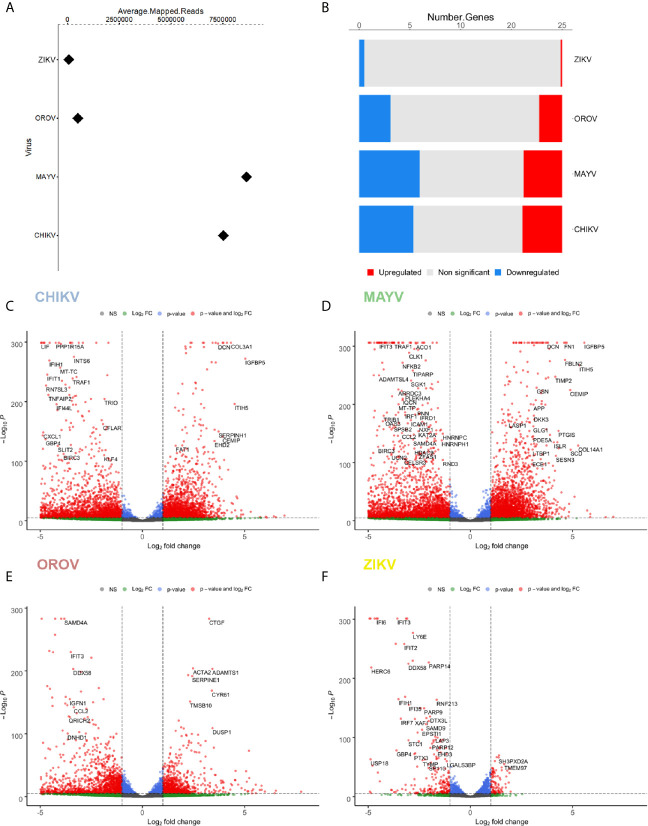
Differentially expressed genes (DEGs) signature for CHIKV, MAYV, OROV, and ZIKV in human primary astrocytes. Signature of DEGs detected in RNA-Seq of infected (n = 3) over uninfected (n=6) cells. **(A)** Relative abundance (average of mapped reads) of viral RNA in hAST infected with CHIKV, MAYV, OROV, and ZIKV. **(B)** Number of up- and down-regulated genes for CHIKV, MAYV, OROV, and ZIKV with statistical significance (p ≤ 0.05 and at least 1 log_2_FC dysregulation over uninfected cells). Volcano plots for the differentially expressed genes (DEGs) for CHIKV **(C)**, MAYV **(D)**, OROV **(E)**, and ZIKV **(F)**. RNA-seq from triplicates were performed from CHIKV, MAYV, and OROV infected cells 18 hpi and ZIKV infected cells 48 hpi. X-axis represents the log_2_ Fold Change of DEG relative to uninfected cells and Y-axis depicts significance. Hpi, hours post infection.

To analyze host cellular genes and pathways modulated upon infection, we performed a transcriptome-wide sequencing of uninfected (n = 6) and infected hAST with each virus (n = 3). Transcriptome analyses were performed at 18 hpi for CHIKV, MAYV, and OROV and 48 hpi for ZIKV, following the infection peak and absence of cytopathic effect for each virus (**Figures 1B, C**). We found an increasing number of RNA-seq reads that mapped on CHIKV and MAYV genomes compared with OROV and ZIKV suggesting that alphaviruses present higher replication rates in hAST (**Figure 2A**). As a reflection of higher titers of viral replication for alphaviruses, we found an increasing number of cellular genes dysregulated by CHIKV and MAYV compared with OROV and ZIKV (**Figure 2B** and **Table S2**).

Briefly, we found 3578, 4104, 2056, and 133 up-regulated genes for CHIKV, MAYV, OROV, and ZIKV infected cells, respectively, in contrast with 4883, 6388, 2802, and 415 down-regulated genes (p ≤ 0.05 and at least 1 log_2_FC dysregulation over uninfected cells) (**Figures 2C–F**). Those results reflect an increasing impact in the modulation of differentially expressed genes (DEGs) that corroborates with the crescent genus-dependent viral replication as follows: flavivirus, orthobunyavirus, and alphaviruses. Altogether, the four viruses interfere with astrocyte gene expression down-regulating a higher number of genes compared with up-regulated genes (**Figures 2C–F** and **Table S2**).

### CHIKV Interferes With Extracellular Matrix, N-Glycosylation, Translation, Cell Cycle, and Interferon Pathways

**Figure 3:**
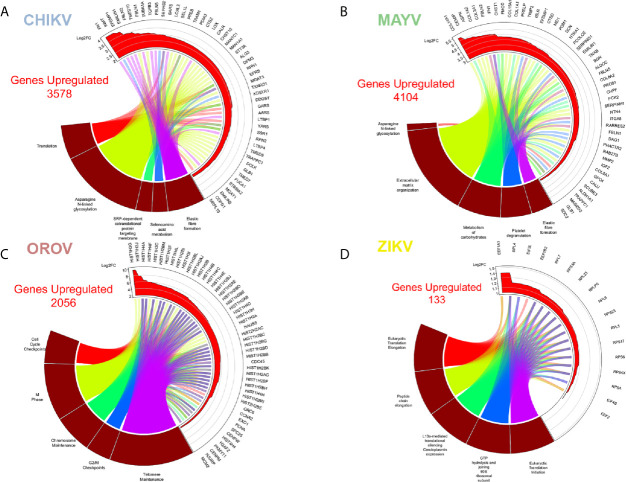
Enriched up-regulated pathways in CHIKV, MAYV, OROV, and ZIKV infected astrocytes. Cell pathways with up-regulated genes in **(A)** CHIKV (18 hpi), **(B)** MAYV (18 hpi), **(C)** OROV (18 hpi), and **(D)** ZIKV (48 hpi) infected cells. Circus plot of Gene Set Enrichment Analysis (GSEA) of the five up-regulated most significantly enriched Reactome pathways (dark red sectors at the bottom of the circus plot) with the 50 associated DEGs. The 20 most significant up-regulated pathways are depicted. In the upper sector of the circus plot the genes were ordered by modulus of log2 Fold Change relative to uninfected cells.

Our reactome analysis showed that CHIKV infected astrocytes presented an enrichment of up-regulated DEGs in pathways related to protein translation, post-translational modification of glycoproteins (N-linked glycosylation and metabolism of aminoacid), remodeling of extracellular matrix, protein translation, and targeting to endoplasmic reticulum (ER) membrane (**Figure 3A** and **Table S3**). The most significant genes associated with those pathways were: extracellular matrix/elastic fiber formation, as fibronectin 1 (*FN1*), fibulin (EGF Containing Fibulin Extracellular Matrix Protein 1, *EFEMP1*; Fibulin 1, *FBLN*), and elastin (Elastin Microfibril Interfacer 2, *EMILIN2*); amino acid activation, including aminoacyl-tRNA synthetases (Seryl-tRNA Synthetase, *SARS*; Bifunctional Aminoacyll-tRNA Synthetase, *EPRS*; Glycyl-tRNA Synthetase 1, *GARS*; Alanyl-tRNA Synthetase 1, *AARS*; Tyrosyl-Trna Synthetase 1, *YARS*); ER associated translation (Translocation Associated Membrane Protein 1, *TRAM1*; Protein Disulfide Isomerase Family A Member 3, *PDIA3*; Calreticulin, *CALR*; KDEL Endoplasmic Reticulum Protein Retention Receptor 1, *KDELR1*); and glycosylation (LEM Domain Containing 3, *MAN1*; Beta-1,4-Mannosyl-Glycoprotein 4-Beta-N-Acetylglucosaminyltransferase, *MGAT3*; Dolichyl-Diphosphooligosaccharide–Protein Glycosyltransferase Non-Catalytic Subunit, *DDOST*; Alpha-1,3-Mannosyl-Glycoprotein 2-Beta-N-Acetylglucosaminyltransferase, *MGAT1*). Translation factors are usually modulated by RNA viruses to promote viral protein production (Au and Jan, 2014). Indeed, the glycosylation of virus envelope proteins are crucial to increase CHIKV virulence and the up-regulation of genes related to asparagine N-linked glycosylation reinforces this hypothesis (Acharya et al., 2015; Lancaster et al., 2016). Cytoskeleton tubulin isoforms predominantly found in differentiated neurologic cells (Tubulin Alpha 1a, *TUBA1A*) and transmembrane vesicular protein involved in traffic (Transmembrane P24 Trafficking Protein 10, *TMED10*) were highly induced by CHIKV (**Figure 3A**), showing the importance of glycosylation pathways not only for envelope proteins, but also in virus assembly and budding.

**Figure 4:**
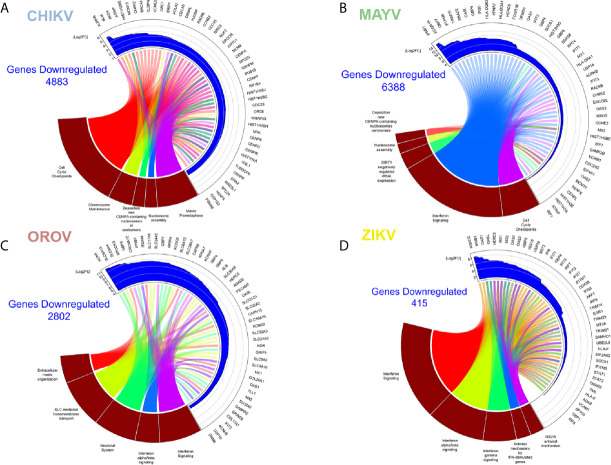
Enriched down-regulated pathways in CHIKV, MAYV, OROV, and ZIKV infected astrocytes. Cell pathways with down-regulated genes in **(A)** CHIKV (18 hpi), **(B)** MAYV (18 hpi), **(C)** OROV (18 hpi), and **(D)** ZIKV (48 hpi) infected cells. Circus plot of Gene Set Enrichment Analysis (GSEA) of the five down-regulated most significantly enriched Reactome pathways (dark red sectors at the bottom of the circus plot) with the 50 associated DEGs. The 20 most significant down-regulated pathways are depicted. In the upper sector of the circus plot the genes were ordered by modulus of log2 Fold Change relative to uninfected cells.

CHIKV down-regulated pathways in astrocytes included: chromosome stability, DNA repair and maintenance; and cell cycle (in special, nucleosome assembly and prometaphase directed steps) (**Figure 4A** and **Table S4**). It is known that viruses always interfere with cell cycle to increase their own replication, promoting virus translation and avoiding apoptosis (Bressy et al., 2019; Caller et al., 2019). In fact, our reactome analysis of down-regulated pathways showed a consistent relationship within cell cycle controlling genes, indicating a possible G2 arrest phenotype (**Figure 4A**). Among the down-regulated genes were: genes from the cell division processes, as centrosome, centromere, and tubulin associated genes (Centrosomal Protein, *CEPs*; Centromere Protein, *CENPs*; and Tubulin, *TUBs*); cell cycle (Cell Division Cycle, *CDCs*); and chromosome stability, such as histones (*HISTs*) and DNA polymerase subunits *(POLs*) (**Figure 4A**). This goes in agreement with previous reports of CHIKV interfering with cell cycle in other cell models (Saxena et al., 2013; Thio et al., 2013). The *Aurora Kinase B* (*AURKB*), a chromosome segregation and cytokinesis promoting gene, was the most down-regulated gene in CHIKV infected cells (**Figure 4A**). Although the role of *AURKB* depletion has been controversially described for flaviviruses (Madejón et al., 2015; Pérez-Olais et al., 2019), its impact remains elusive for alphaviruses; nonetheless, it could be speculated as a reinforcer of G2 arrest.

### MAYV Dysregulates the Same Profile of CHIKV Associated Pathways, With Prominent Down-Modulation of Interferon Response Genes

MAYV infection led to an enrichment and up-regulation of elastic fiber formation and N-glycosylation pathways, as in CHIKV infection; carbohydrates metabolism, platelet degranulation, and extracellular matrix organization (**Figure 3B**). The gene interactome modulated by MAYV is highly heterogeneous compared with CHIKV. Nonetheless, many genes related to elastic fiber/extracellular matrix pathways as integrins (*ITGA8*), fibronectin (*FN1*, also highly up-regulated in CHIKV infection), collagens (*COLs*), and metalloproteinases (*MMP2*) were up-regulated by MAYV (**Figure 3B**). Metalloproteinases are involved in extracellular matrix degradation and are associated with neurological outcomes in viral infections (Savarin et al., 2011; Shukla et al., 2016; Xing et al., 2017; Barkhash et al., 2018; Song et al., 2018; Chopra et al., 2019).

MAYV shares common down-modulated pathways with CHIKV, such as as Type-I interferon signaling (**Table S2**), nucleossome assembly and formation, and cell cycle (**Figure 4B** and **Table S4**). Reactome analysis showed two main clusters: (i) one related to interferon signaling and innate immunity, and (ii) another one related to pathways associated to cell cycle arrest, and repression of DNA replication and cell division (**Figure 4B**). MAYV and CHIKV, both alphaviruses, down-regulated common genes related to cell cycle and chromosome stability, such as *CENPs*, *HISTs*, and *CDCs* (**Figures 4A, B**). Indeed, interferon (*IFNB1*), interferon stimulated genes—ISGs (Interferon Induced Protein With Tetratricopeptide Repeats, *IFITs*; Radical S-Adenosyl Methionine Domain Containing 2, *RSAD2*; MX Dynamin Like GTPase 1, *MX1*; 2’-5’-Oligoadenylate Synthetase, *OASs*; Tripartite Motif Containing, *TRIMs*) and agonists of interferon response (DExD/H-Box Helicase 58, *DDX58*; Interferon Regulatory Factor 7, *IRF7*; Interferon Regulatory Factor 1, *IRF1*) were represented among the most down-regulated genes by MAYV, with higher fold change values, up to 12 (*IFNB1*) (**Figure 4B**).

### OROV Infection Interferes With Ion Transport, Neuronal Synapses Regulation and Type-I Interferon Pathways, Contributing to Neuropathogenesis

OROV infection in human astrocytes, contrasting with alphaviruses infection, up-regulated pathways related to cell cycle, cell division, DNA maintenance and replication, and extracellular matrix remodeling (**Figure 3C** and **Table S3**). The opposite direction of pathways regulated by Alphavirus and Orthobunyavirus demonstrates the complexity and diversity in host factors modulation and may represent different strategies of virus replication. The pathway reactome corroborates the overall tendency in OROV infection, up-regulating pathways related to DNA condensation (mainly through the core H2, H3, and H4 histones), cell cycle checkpoints and G2 arrest, and telomere maintenance (**Figure 3C** and **Table S3**). Some proteins from related orthobunyaviruses were already demonstrated to have nuclear localization, interfering with cell cycle and hampering global cellular transcription through histone modification (Copeland et al., 2013; Gouzil et al., 2017). The genes in the reactome corroborated this hypothesis, as many histones, *CENPs*, and other G2/M step related proteins were up-regulated. The G2/M arrest could increase the availability of capped-mRNAs, which serves as primer donors for bunyaviruses translation: a process named cap-snatching. Corroborating our findings, and in contrast with bunyaviruses, alphaviruses can cap their own RNAs using nsP activities (Hopkins and Cherry, 2013) (**Figure 3C**).

Overall, the pathways found down-regulated by OROV in astrocytes corroborate the dysregulation of interferon response; highlighting genes with antiviral activities (*RSAD2*, also known as viperin; *OASL*; *OAS1*; *MX1/2*; *IFIT2*). However, unlike alphaviruses, OROV infection also down-regulated extracellular matrix remodeling genes [such as SPARC (Osteonectin), Cwcv And Kazal Like Domains Proteoglycan 3, *SPOCK3*, and Integrin Subunit Alpha M, *ITGAM*] and collagen metabolism pathways (Collagen Type XVII Alpha 1 Chain, *COL17A1*, and Collagen Type XX Alpha 1 Chain, *COL20A1*) (**Figure 4C** and **Table S4**). Moreover, OROV infection also down-modulates ion channels, transmembrane solute carriers, and synapses regulation pathways that play crucial roles in astrocytes functions. Genes related to potassium channels (Potassium Voltage-Gated Channel, *KCNs*, and Hyperpolarization Activated Cyclic Nucleotide Gated Potassium Channel 4, *HCN4*) and solute transporters from the family of Solute Carrier (*SLCs*) were the most representative clusters (**Figure 4C**) down-modulated by OROV. Recently, it was demonstrated that K^+^ channels are up-regulated and therefore required for endosomal trafficking and high infectivity of bunyaviruses, corroborating our findings (Hover et al., 2016; Hover et al., 2018). SLC family of transporters includes Excitatory Amino Acid Transporter 1 (*EAAT1*, also known as*SLC1A3*/*GLAST*) and Excitatory Amino Acid Transporter 2 (*EAAT2*, also known as *SLC1A2*/*GLT1*), both glutamate transporters whose dysfunction have been implicated in increasing neurotoxicity driven by accumulation of extracellular glutamate (Legay et al., 2003; Wang et al., 2003; Moidunny et al., 2016). Those pathways, together with down-regulation of Type-I interferon signaling, could increase the neuropathological manifestations of OROV infection.

### ZIKV Up-Regulated Eukaryotic and Viral Translation Pathways, and Down-Regulated Type-I Interferon Signaling Proteins

ZIKV infection modulated the least number of genes (133 up- and 415 down-regulated, respectively) compared with the other viruses. This observation is in agreement with the lower replication rates expected for flaviviruses compared with alphaviruses or orthobunyaviruses (**Figures 1B–D**). However, it is important to note that Asian derived strains (as the Brazilian one used here) have usually lower virulence and replication kinetics compared to African strains (Simonin et al., 2016; Hamel et al., 2017). Nonetheless, the most enriched up-regulated pathways upon ZIKV infection were viral mRNA translation, translation elongation, aminoacid synthesis, ribosomal assembly, and cap-dependent translation (**Figure 3D** and **Table S3**); all related to host translation machinery. All up-regulated genes were either eukaryotic initiation (*EIFs*) or elongation factors (*EEFs*), and ribosomal proteins (*RPSs*and *RPLs*) (**Figure 3D**). Most of the pathways down-regulated by ZIKV in astrocytes were related to interferon-induced antiviral innate immune response (**Figure 4D** and **Table S4**). The clusterization of the pathways classified them into type I and II interferon signaling, and antiviral interferon-responsive genes (**Figure 4D**). Transcriptional factors related to interferon pathways were also down-modulated (*IRFs* and Signal Transducer And Activator of Transcription, *STATs*), reinforcing the relevance of ISGs in controlling ZIKV infection. As demonstrated by other cellular models, most genes down-regulated by ZIKV were ISGs: *RSAD2*, *IFITs*, Guanylate Binding Protein (*GBPs*), *MXs*, and *OASs* (**Figure 4D**). Cytoplasmatic RNA sensor (*DDX58*, also known as Retinoic Acid Inducible Gene I, *RIG-I*) and classical interferon induced antiviral genes that direct block virus replication at several steps (SAM And HD Domain Containing Deoxynucleoside Triphosphate Triphosphohydrolase 1, *SAMHD1*: RNA virus replication; *TRIMs*: virus uncoating; and Bone Marrow Stromal Cell Antigen 2, *BST2*: virus budding, respectively) were also down-modulated by ZIKV infection. All this scenario suggests that ZIKV establishes stable infection in astrocytes counteracting and down-modulating interferon pathway at several steps.

### Viral Infection Leads to an Overall Down-Regulation of Innate Immunity Genes in Astrocytes

**Figure 5:**
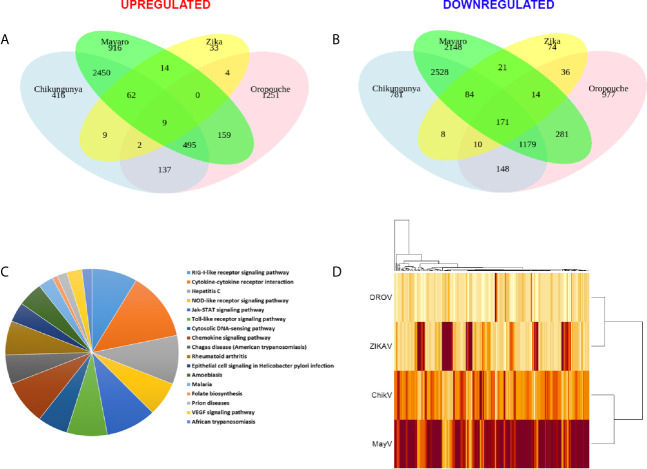
Common differentially expressed genes (DEGs) signature for CHIKV, MAYV, OROV, and ZIKV in human primary astrocytes. Signature of DEGs detected in RNA-Seq of infected (n = 3) over mock uninfected (n = 6) hAST cells. Venn diagram showing the quantity of common and exclusive up-regulated **(A)** and down-regulated **(B)** genes found in the RNA-Seq dataset. Only DEGs with at least 1 log_2_ Fold Change and an adjusted P value ≤ 0.05 are shown. **(C)** Gene ontology by biological processes of 171 common down-regulated genes by all viruses (CHIKV, MAYV, OROV, and ZIKV). **(D)** Heatmap of the common up-regulated (9) and down-regulated (171) genes among all viruses here included. Dendrogram of Euclidian hierarchical clusterization among the four viruses are shown. Color scale represents baseMean (estimative of abundance) of up-regulated and down-regulated genes (light yellow represents lower and brown represents higher abundance).

We performed RNA-seq integration to identify common pathways modulated by the four viruses. Only 9 up-regulated and 171 down-regulated DEGs were co-modulated by CHIKV, OROV, MAYV, and ZIKA (**Figures 5A, B**, respectively and **Table S5**). As expected, CHIKV and MAYV shared a higher number of up- (2,450) and down- (2,528) regulated genes among themselves compared with other viruses analyzed, suggesting a signature for alphaviruses (**Figures 5A, B**). Gene ontology analysis of the 171 common down-regulated genes indicated that most of those genes belongs to pathways related to virus RNA sensing (*RIG-1*), interferon signaling (including ISGs), cytokines and chemokine signaling, JAK-STAT (Janus Kinase-STAT), and Toll receptors signaling, suggesting a global modulation of innate immune response and inflammatory response in primary astrocytes infected by RNA viruses (**Figure 5C** and **Table S5**). The alphaviruses CHIKV and MAYV also cluster together when analyzing only the common dysregulated genes for all viruses, as expected (**Figure 5D**). Deeper analysis of the virus RNA sensing and interferon signaling pathways down-modulated by the four viruses identified antiviral genes triggered by interferon stimulation (ISGs), such as: *ISG15*, *MX1-2*, *DDX58*, CT And RLD Domain Containing E3 Ubiquitin Protein Ligase 5 (*HERC5*), *OAS*, Interferon Induced Protein With Tetratricopeptide Repeats (*IFIT*), Adenosine Deaminase RNA Specific (*ADAR*), *SAMHD1*, Interferon Induced Transmembrane Protein 1 (IFITM1), and *TRIMs*; all those already described for other RNA and DNA viruses infections.

**Figure 6:**
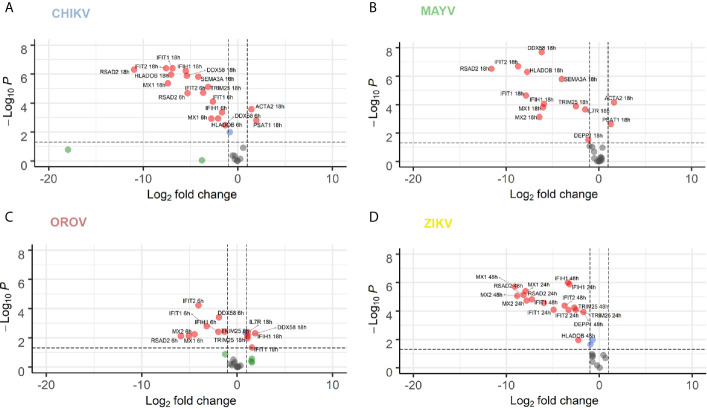
RT-qPCR validation of 16 selected DEGs co-modulated by CHIKV, MAYV, OROV, and ZIKV. hAST cells were infected with MOI 1 of CHIKV **(A)**, MAYV **(B)**, OROV **(C)** at 6 and 18 hpi and ZIKV **(D)** at 24 and 48 hpi and RT-qPCR was performed at indicated time points and compared to uninfected cells (n = 3 for each time point and condition). Data denotes p-values (y-axis) and mean fold change (x-axis) of infected cells relative to uninfected cells for 16 DEGs selected from commonly modulated genes in RNA-Seq dataset. Gene expression was normalized by endogenous *HPRT1* levels. Red dots represent DEGs with at least 1 log2 Fold Change and P value ≤ 0.05. Blue dots represent DEGs with only P value ≤ 0.05. Green dots represent DEGs with at least 1 log2 Fold Change. Grey dots represent non-significant DEGs.

We performed RT-qPCR of 16 common genes dysregulated for all viruses in order to validate our RNA-seq data (**Figure 6**). We selected genes belonging to cytoskeleton and motility (Actin Alpha 2, *ACTA2*) (Rockey et al., 2019), autophagy (Decidual Protein Induced By Progesterone, DEPP1), L-serine synthesis (Phosphoserine Aminotransferase 1, *PSAT1*) (Jiang et al., 2020), inflammatory response (Interleukin 7 Receptor, *IL7R*, and Major Histocompatibility Complex, Class II, DO Beta, *HLA-DOB*) (Inchley et al., 2013; Alsadeq et al., 2018), axonal guidance and neuropathology (Semaphorin 3A, *SEMA3A*) (Niclou et al., 2003; Yang et al., 2019), interferon induction (*DDX58*, Interferon Induced With Helicase C Domain 1, *IFIH1*, *TRIM25*, and *IRF7*), and interferon responsive/agonist genes with antiviral activity (*IFIT1*, *IFIT2*, *MX1*, *MX2*, *ISG15*, and *RSAD2*) (García-Sastre, 2017; Schoggins, 2019).

Overall, the majority of genes tested by RT-qPCR confirmed the dysregulation in expression found in our RNA-Seq data. To validate our findings, we also perform a kinetics of DEGs during virus infection at increasing time points. Most of the tested genes presented an increasing time-dependent down-regulation in later time points for each virus infection (6 and 18 hpi for CHIKV, MAYV, and OROV; 24 and 48 hpi for ZIKV) (**Figures 6A–D**, respectively). The only exceptions found were *DDX58*, *IFIT*, and *IFIH1*, that were down-modulated at early times of OROV infection and lose significance at later points. *PSAT1* and *ACTA2* were up-regulated in response to all viral infections, with higher induction in cells infected with CHIKV and MAYV at 18 hpi. Those genes are related to cell cytoskeleton and invasion, suggesting the virus dependence of intracellular trafficking to sustain replication. ISGs and immune response genes such as *IFIT1*, *IFIT2*, *IFIH1*, *MX1*, *MX2*, and *RSAD2* were highly down-regulated in response to all viruses at later time points of infection, confirming our transcriptome data. The intracellular virus RNA sensor *DDX58* (*RIG-I*) was down-regulated by all viruses, regardless the virus family, showing the central importance of this pathway in recognize viruses in infected cells and stimulate IFN and ISGs. Taken together, those results corroborate our transcriptome analysis showing that viruses from different families, such as CHIKV, MAYV, OROV, and ZIKV, interfere with antiviral and interferon mediated immune response to stablish an efficient and persistent infection in human primary astrocytic cells.

## Discussion

Arboviruses from *Togaviridae*, *Peribunyaviridae*, and *Flaviviridae* families account for the most epidemics and public health system burden in tropical countries, mainly in South and Central Americas. Although the most common manifestations of acute infection involve self-limiting febrile illness with mild to moderate symptoms, in some cases those viruses can invade CNS and generate severe neurological disorders, some of them with mimicking phenotypes of inflammatory autoimmune nervous system diseases. Even so, the mechanisms of neuropathogenesis and host interaction remain poorly understood for most of them. Here, we presented a comprehensive genome-wide transcriptome analysis of human primary astrocytes infected with CHIKV, MAYV, OROV, or ZIKV.

The capacity to infect and sustain productive infection in astrocytes was previously demonstrated for ZIKV (Hamel et al., 2017; Kozak et al., 2017; Meertens et al., 2017; Chen et al., 2018; Stefanik et al., 2018; Sher et al., 2019). However, to date there was no report of the same capacity for CHIKV, MAYV, and OROV in human cells. Those viruses were not only capable of infecting but demonstrated a more prominent viral production than ZIKV (**Figures 1C, D**, and **2A**). Those findings demonstrated that arthritogenic alphaviruses and OROV have also neurotropic potential like already described for encephalic alphaviruses (Ronca et al., 2016) and encephalic orthobunyaviruses (Evans and Peterson, 2019). Our RNA-Seq data showed an greater number of DEGs and different cellular pathways modulated by viruses presenting higher viremia since the beginning of infection (CHIK, MAYV e OROV), compared with lower replication rates of ZIKV in human primary astrocytes. Indeed, viruses from the same family, such as CHIKV and MAYV (*Togaviridae*), dysregulate a higher number of common genes and cellular pathways compared to OROV and ZIKV that belong to *Peribunyaviridae* and *Flaviviridae* families, respectively. Those results suggest that the virus genome RNA polarity (positive or negative) and replication strategy could differentially interfere with cellular pathways. However, the integration analysis showed a common signature of DEGs by those different viruses, reflecting the down-regulation of antiviral innate immune response and interferon pathway. This observation suggests that to stablish an efficient and productive infection in human primary astrocytes, viruses from different families need to counteract the cellular and immune response against the virus replication (**Figure 5**). *DDHX58* (*RIG-I*), a cytosolic dsRNA-sensing protein, was consistently down-regulated among all viruses. Besides, *IFN-β* was the second most commonly down-regulated gene to all viral infections (**Table S5**). Transcription factors that control interferon induction such as IRFs, mainly IRF-7, was also consistently down-regulated. Since the upstream RIG-I along with IRFs were down-regulated, it is reasonable to estimate the suppression in *IFN-β* and ISGs expression (**Figure 5** and **Table S5**). However, other RNA-Seq studies in mice (Wilson et al., 2017) and in human cohort (Soares-Schanoski et al., 2019) showed that CHIKV infection led to a global up-regulation in innate immunity and pro-inflammatory genes. The same was observed in neuroprogenitors cells infected with ZIKV (Lima et al., 2019). In case of ZIKV, though induced in neuroprogenitor cells, interferon, innate immunity, and inflammation were repressed in microglia (Tiwari et al., 2017) and dendritic cells (Sun et al., 2017), indicating cell type-specific dynamic in regulation of antiviral response. Aside of cell or tissue specific responses, another hypothesis that could explain contradictory innate immunity regulation among the datasets is the time-dependent effect on gene expression. In general, previous studies showed an induction of IFN pathways triggered by RIG-I recognition of virus double strand RNA structures at early points of RNA viruses infection, followed by antiviral and ISGs stimulation. Our results showed a common down-regulation of this pathway in later steps of CHIKV, MAYV, OROV, and ZIKV infection, possibly as a viral escape response to innate immune antiviral genes, allowing their replication in human astrocytic cells. A recent study using Vesicular Stomatitis Virus (VSV) as model proposed that a social evolution dictates the innate immunity evasion (Domingo-Calap et al., 2019). According to this model, IFN suppression is a costly mechanism, but it is evolutionary necessary to establish an efficient and productive infection. Due to high infectivity (about 80% of cell population) in astrocytes in a relative short time span (**Figure 1B**), it is likely that innate immunity suppression is occurring in early moments after infection. Among the various ISGs found, *IFITs* and *MXs* were also consistently down-regulated (**Figure 6**). From those, *RSAD2*, also known as Viperin, is a well characterized antiviral protein that already was demonstrated to restrict ZIKV (Panayiotou et al., 2018) and CHIKV (Teng et al., 2012), but its antiviral activity against MAYV and OROV was never described. Interestingly, in opposition to our results, in another study was demonstrated that *RSAD2* as wells as IFN response is induced upon ZIKV infection in mice astrocytes (Lindqvist et al., 2016); however, *RSAD2* regulation and its capacity to restrict viral replication in human astrocytes remain to be investigated.

Eukaryotic translation factors and ribosomal proteins were up-regulated upon ZIKV (**Figure 3D**), which reflects the viral recruitment of host translation machinery for viral protein synthesis (Walsh et al., 2013; Li, 2019). CHIKV and MAYV both up-regulated N-glycosylation machinery (**Figures 3A, B**), which, coupled with the up-regulation of translation machinery, suggests a multiple-step retargeting of host cell machinery towards viral protein synthesis and maturation (Acharya et al., 2015; Lancaster et al., 2016). Moreover, metalloproteinases, collagen and integrin genes were found up-regulated by CHIKV and MAYV infection but down-regulated in response to OROV infection (**Figure 4C**), suggesting opposite regulation of extracellular matrix and adhesion proteins between the two viral families. Regulation of CNS extracellular matrix by astrocytes is fundamental to neurogenesis, neuronal migration, and formation of new synapses (Cope and Gould, 2019); therefore, any unbalancing in the processes could lead to neuropathological effects.

Another interesting divergent regulation was observed between alphaviruses and OROV. Cyclin Dependent Kinase 1 (*CDK1*) and cyclins (*CCNs*) A, B, and E were down-regulated in CHIKV and MAYV infection, while they were up-regulated in OROV infection (**Table S2**). Those results suggest a G1 cell cycle arrest induced by alphaviruses, and a G2/M arrest for OROV. G1 arrest was already described to be beneficial to viral production (Lundberg et al., 2018; Wang et al., 2018), which corroborates the overall recruitment of host translation and glycoprotein processing machineries in order to favor alphavirus production. Rift Valley Fever Virus (RVFV), a bunyavirus from *Phleboviridae* family, induces G2 cell cycle arrest for optimal cap-snatching and viral replication (Hopkins et al., 2013). It is possible that OROV induces the same cell cycle arrest in G2, what should be further investigated, since cap-snatching was never proved for OROV, but it is common for other bunyaviruses. OROV infection also down-regulated many membrane transporters and ion channels (**Figure 4C**). From those, *SLC1A2*/*EAAT2* (see **Table S2**) is implicated in glutamate uptake, suggesting that its down-regulation lead to accumulation of extracellular glutamate, which is often associated to neurotoxicity (Legay et al., 2003; Wang et al., 2003; Moidunny et al., 2016). We previously demonstrated the down-regulation of this gene in OROV infected HuH-7 cells and its possible association with upregulation of *miR-217* (Geddes et al., 2018). Taken together, that suggests that *EAAT2* might be a regulated target in OROV infected cells that could explain the neuropathogeny associated to the virus.

In this study, we provided an integrated genome-wide transcriptome analysis of human primary astrocytes, crucial cells responsible for a variety of functions in the CNS, infected with the emergent and clinically alarming human pathogens CHIKV, MAYV, OROV, and ZIKV. To our knowledge, this is the first study to present a transcriptome for MAYV and OROV, as well as first to present CHIKV transcriptome in cells from CNS. This work shed a light upon the mechanisms and viral-host interactions in the neuropathologies associated to those widespread human arboviruses regarding nervous system outcomes.

## Data Availability Statement

The datasets presented in this study can be found in online repositories. The names of the repository/repositories and accession number(s) can be found in the article/**Supplementary Material**.

## Author Contributions

Conceptualization, VG and RA. Methodology, VG, OB, LR, JS, AV, and RA. Validation, VG, OB, and LC. Formal analysis, OB, FM, and RS. Investigation, VG, LC, FC, AG, AL, CF, and LD. Resources, CF, EN, AT, IA-M, SA-L, LR, JS, AV, and RA. Data curation, OB. Writing—original draft, VG, OB, and RA. Writing—review and editing, VG, OB, LC, FC, RS, IA-M, SA-L, LR, JS, AV, and RA. Visualization, VG, OB, AV, and RA. Supervision, AV and RA. Project Administration, AV and RA. Funding Acquisition, AV and RA. All authors contributed to the article and approved the submitted version.

## Funding

This research was supported by FINEP (grant no. 01.16.0078.00) and the European Union’s Horizon 2020 research and innovation program through the ZIKAlliance project (grant agreement no. 734548). VG is supported by CAPES under the grant number: 1545985. RA is supported by CNPQ 312688/2017-2, 439119/2018-9; FAPERJ 202.922/2018. AV is supported by CNPq (303170/2017-4) and FAPERJ (26/202.903/20). LC is supported by RABICO/CAPES Project 88887.333817/2019-00. IA-M is supported by CNPq (436933/2018-7) and FAPERJ (203.225/2017).

## Conflict of Interest

The authors declare that the research was conducted in the absence of any commercial or financial relationships that could be construed as a potential conflict of interest.

## Supplementary Material

Supplementary Table 1 Oligonucleotides used in this study.

Supplementary Table 2 All DEGs found in transcriptome for each virus.

Supplementary Table 3 50 most up-regulated genes for each virus used in Circoplot.

Supplementary Table 4 50 most down-regulated genes for each virus used in Circoplot.

Supplementary Table 5 Common DEG among CHIKV, MAYV, OROV, and ZIKV.
